# Effectiveness of sorafenib in treating intermediate-stage hepatocellular carcinoma patients refractory to transarterial chemoembolization

**DOI:** 10.1186/s12885-024-13199-1

**Published:** 2024-11-28

**Authors:** Reham Ashour, Eman Rewisha, Mohamed AKL Rady, Sally Waheed Elkhadry, Heba Abdelhalim, Mohamed Atef

**Affiliations:** 1 Department of Hepatology and Gastroenterology, National Liver Institute, Menoufia University, Shibin El-Kom, 32511 Egypt; 2 Department of Epidemiology and Preventive Medicine, National Liver Institute, Menoufia University, Menoufia, Shibin El Kom City, 32511 Egypt; 3 Department of Diagnostic Medical Imaging and Interventional Radiology, National Liver Institute, Menoufia University, Shibin El-Kom, 32511 Egypt

**Keywords:** Hepatocellular carcinoma, Sorafenib, Transarterial chemoembolization, Refractorness, Intermediate-stage patients

## Abstract

**Background:**

Switching to systemic therapy after transarterial chemoembolization (TACE) refractoriness is more inclined to preserve liver function and decrease disease progression. Hence, we conducted a comparison between the advantages of sorafenib therapy and the continuation of TACE in patients with intermediate-stage hepatocellular carcinoma (HCC) who developed TACE refractoriness.

**Methods:**

This retrospective cohort work involved 1,200 patients with HCC who received TACE therapy at our institution between January 2018 and December 2022. Out of these, a total of 436 participants were determined to be resistant to TACE treatment throughout their clinical progression. Out of them, 271 were finally included and categorized into two groups: (1) patients who shifted from TACE to sorafenib, and (2) patients who maintained TACE treatment. The study assessed the overall survival (OS) and time to disease progression (TTDP) of patients who were resistant to TACE, comparing both groups based on when they achieved Child-Pugh C or acquired advanced-stage HCC.

**Results:**

Following confirmation of refractoriness to TACE therapy, 163 opted to continue with TACE (TACE group), whereas 108 shifted to sorafenib treatment (sorafenib group). The median TTDP was 23.36 months, while the median OS was 25.3 months, in the sorafenib group, and 11.6 and 14.2 months, correspondingly, in the TACE group (*p* = 0.0001).

**Conclusion:**

Switching to sorafenib treatment significantly improved OS and TTDP in patients with intermediate-stage HCC who were refractory to TACE. These finding highlights sorafenib’s potential as an effective alternative for managing disease progression in patients unresponsive to TACE, offering a valuable treatment option in this challenging clinical scenario.

**Supplementary Information:**

The online version contains supplementary material available at 10.1186/s12885-024-13199-1.

## Background

Transarterial chemoembolization (TACE) is the primary therapeutic approach suggested by the Barcelona Clinic Liver Cancer (BCLC) staging system for those suffering from hepatocellular carcinoma (HCC) who are at an intermediate stage (BCLC B) [1–3]. However, in clinical settings, TACE is beneficial for only 50–60% of those with BCLC stage B, necessitating repeated sessions to achieve maximum tumor reduction. Despite that, it does not fully cure the majority of HCC cases [4]. The deterioration of liver function and high recurrence rates are the primary factors limiting TACE’s efficacy, the latter resulting from residual tumor growth stimulated by the angiogenic response to TACE-induced hypoxia [4].

Furthermore, the likelihood of tumor metastasis and recurrence is increased by repeated TACE, which also promotes resistance. Additionally, it is correlated with a greater risk of adverse effects including liver damage [3–5]. Due to these concerns, TACE refractoriness has garnered significant interest. TACE failure/refractoriness was initially proposed in 2010 by the Japan Society of Hepatology and updated in 2014 and 2021 [6–8].

The International Expert Panel on Interventions in Hepatocellular Carcinoma (EPOIHCC) defined TACE refractoriness as no responses following three or more TACE procedures performed on the same location within six months [9, 10]. Subsequent research revealed that TACE refractoriness significantly affects prognosis [11–13]. Additionally, TACE refractoriness/failure has been recognized in guidelines from various organizations, including the American Association for the Study of Liver Diseases (AASLD) and the Asian Pacific Association for the Study of the Liver (APASL) [14, 15]. The European Association for the Study of the Liver (EASL) and the recommendations of the European Organization for Research and treatment of Cancer (EORTC) suggest providing refractory patients with the most suitable alternative therapy option available at the same stage [16].

The updated 2022 BCLC guidelines recommend that for HCC individuals within the BCLC B category who develop TACE refractoriness, the therapeutic approach should be shifted towards systemic therapies. These include targeted therapies or immune checkpoint inhibitors [17]. Among the targeted therapies, Sorafenib, a multikinase inhibitor, is suggested as a treatment option for intermediate-stage HCC individuals who are TACE refractory [6, 16, 17]. Despite the importance of optimizing treatment approaches for this critical disease, there have been few studies comparing the effectiveness of shifting to sorafenib versus continuing repeated TACE among individuals with intermediate-stage HCC who were TACE refractory [5, 18]. Hence, the purpose of this work was comparing the clinical outcomes of sorafenib shifting with ongoing TACE among individuals with intermediate-stage HCC treated at our institution and recognized TACE-refractory.

## Methods

One thousand, two hundred patients diagnosed with intermediate-stage HCC who had the initial TACE at our institution and underwent recurrent TACE were identified retrospectively. Data was retrieved from the medical records at the National Liver Institute, Menoufia University, Egypt, between January 2018 and December 2022. Among these, 271 were diagnosed as TACE-refractory and further allocated into two groups: Patients who either (1) persisted with TACE treatment after being classified as TACE-refractory (TACE group) or (2) shifted from TACE to sorafenib treatment (sorafenib group). They were chosen depending on the specified criteria for inclusion during the initial TACE: (1) Diagnosis of HCC depending on pathological examinations or in accordance with the current practice recommendations of the AASLD and the EASL [19, 20], (2) intermediate-stage HCC as determined by the BCLC staging system [1], and (3) a Child-Pugh score of ≤ 7. So, out of 1200 patients, At the time of the first TACE, we did not include those with any of the subsequent conditions: (1) early-stage HCC classification; (2) HCC at an advanced stage, characterized by the existence of macroscopic vascular infiltration and/or metastases outside the liver; (3) Child-Pugh scores of 8 or more; and (4) insufficient data or patients who were not available for further follow up. Therefore, the final group consisted of 687 patients.

From the final study group, 436 were diagnosed as TACE-refractory, with 271 ultimately enrolled. Exclusions were made for those with Child-Pugh scores of 8 or more, advanced-stage HCC, those who received only best supportive care, those who underwent alternative systemic therapies, or those who were lost to follow-up. The sample size of 271 patients was not pre-determined but was defined by the number of patients who met the inclusion criteria (i.e., diagnosed as TACE-refractory) during the study period. This sample size provides adequate power (80%) to discover significant differences between the two treatment methods that can be generalized, considering the entire population of TACE-refractory patients throughout this timeframe.

Each participant submitted a written informed consent for their therapy, and the work had been permitted by the Ethics Committee of the Institutional Review Board of the National Liver Institute, Menoufia University (NLI IRB 00298/2022).

### Definition of refractoriness of TACE

According to the 2010 JSH Consensus Guidelines [6] TACE refractoriness was recognized utilizing radiological and tumor marker criteria as follows:

#### Radiological Criteria

1. **Intrahepatic lesion**: Two or more successive TACE sessions showing incomplete necrosis (viable lesion > 50%) of intrahepatic lesions, or two or more consecutive appearances of new or recurring liver lesions, that is evident on response evaluation CT or MRI imaging.
2. **Detection of macroscopic vascular invasion**.
3. **Evidence of extrahepatic Spread**.

#### Tumor marker criteria

##### AFP increase

An increase in the tumor marker AFP greater than 20% from the pre-TACE baseline level, even immediately following a TACE session, was used to indicate ongoing tumor activity and resistance to the treatment.

### Technique and Strategy for TACE and sorafenib

A. **Patients’ preparation for TACE**: Participants abstained from eating overnight and hospitalized in the morning for their procedure, where they were administered prophylactic antibiotics and anti-emetics.
B. **Embolization technique**: All TACE procedures were conducted using the Interventional Cath-LAB–configured FD20 AlluraClarity C machine at our interventional radiology unit. The local analgesia was administered, and then the Seldinger method was employed to get to the common femoral artery (CFA) by puncturing the femoral artery. A 6-French vascular sheath was inserted into the CFA using a 0.035-inch Terumo Guide-wire. Using guidance of fluoroscopy, a 5-French Cobra catheter (C2 Boston Scientific) was inserted into the aorta. Angiographic examinations were conducted on the celiac trunk, common hepatic artery and superior mesenteric artery (SMA) to determine all the blood vessels supplying the HCC. Microcatheters were used to selectively cannulate the arterial branches supplying the tumor in order to perform TACE and improve the maintenance of the non-tumoral tissues from the liver around it. An emulsion of lipiodol with either doxorubicin or cisplatin, both of which are anticancer drugs, were administered through the tumour-feeding artery, in cases where the response was insufficient following the previous TACE, adjustments to the chemotherapy agents were allowed. Following the administration of the lipiodol and anticancer drug combination, gelatin sponge particles (Gelfoam) were introduced to fully block the tumour-feeding branch. The endpoint of embolization was determined by the abscence of tumor enhancement observed during hepatic arteriography immediately after chemoembolization.
C. **For patients switched to sorafenib**, oral sorafenib dosage was 400 mg twice a day. Reduction of the sorafenib dose (400 mg either once day or on every other day) and discontinuation were permitted, depending upon the nature and degree of adverse effects. Drug-related toxicities were used to evaluate dose reduction and treatment cessation. Sorafenib therapy was continued until there was severe toxicity or a definite clinical progression of the disease.
D. **All participants underwent regular follow-up examinations** to assess the radiological responses of the tumour every two to three months. The evaluation of radiological response involved contrast-enhanced triphasic CT or dynamic MRI utilizing the modified Response Evaluation Criteria in Solid Tumors (mRECIST) criteria [21]. Additionally, serum α-fetoprotein (AFP) levels were monitored as part of the assessment process.

## The therapeutic efficiency of Sorafenib versus TACE after TACE refractoriness

The therapeutic advantage of switching from TACE to sorafenib was assessed using the “OS” the indicated time span from a determination of TACE-refractoriness to death or the final follow-up.

The time to advanced stage, the time to liver dysfunction, and the TTDP were also recognized. The time from the diagnosis of TACE-refractory until the progression of a Child-Pugh C disease was considered the time to liver dysfunction. The censoring date was determined as the most recent day on which a Child-Pugh score was evaluated, for the patients were not determined to be Child-Pugh C. The time interval between the diagnosis of TACE-refractory and the radiological diagnostic of advanced-stage HCC was the “time to advanced stage.” For patients who did not have advanced-stage HCC, the last radiological evaluation date was considered the censoring date. The term “TTDP” referred to the time from the determination of TACE refractory disease to the emergence of Child-Pugh C or advanced-stage HCC. The last date on which the progression status was appropriately evaluated was defined as the censoring date.

The time to tumor progression (TTP), was also evaluated. It was recognized as the time from the treatment with sorafenib or TACE after the diagnosis of TACE-refractory until the radiological diagnosis of progression by using mRECIST criteria. For patients who showed no progression, the censoring date corresponds to the date of the most recent radiological evaluation.

### Statistical analysis

Both numerical and categorical variables were collected and analysed. Quantitative parameters had been displayed as mean, standard deviation, and range, while qualitative parameters had been displayed as frequencies and percentages. Categorical parameters had been contrasted utilizing the χ2 test. Nonparametric testing (the Mann-Whitney U test) was used for group comparisons. All tests were statistically significant at *p* < 0.05. Follow-up analysis was discontinued at the end of December 2022. Univariate analysis was used to find characteristics that predict survival. The Kaplan-Meier test had been utilized to compare survival curves, with the log-rank test applied. All parameters with *p* < 0.05 were involved in a multivariate analysis to examine their influence as independent predictors. Cox regression was utilized to perform multivariate analysis. Survival was calculated for participants by requesting either their living status, their death time or last follow up. The statistical analyses had been performed employing the SPSS (Statistical Package for Social Science) software, (BM Corp. Released 2013. IBM SPSS Statistics for Windows, Version 22.0. Armonk, NY: IBM Corp).

## Results

**Fig. 1:**
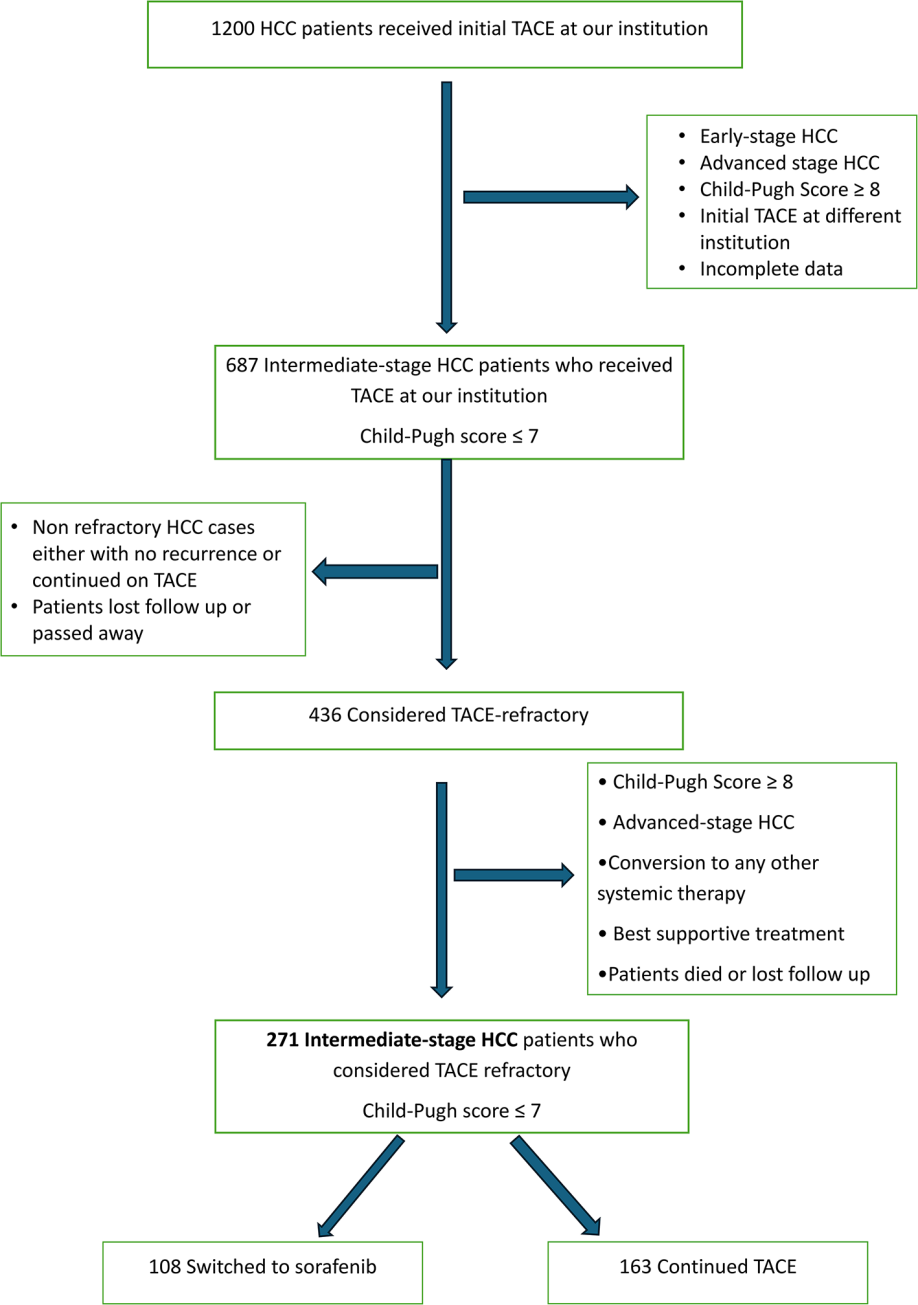
Study flowchart

We evaluated a cohort of 687 individuals suffering from intermediate-stage HCC who had their first TACE treatment at our institution (Fig. 1). throughout the period of follow-up, 436 had been considered TACE-refractory, of which 271 were ultimately included after excluding those with Child-Pugh scores of ≥ 8, advanced-stage HCC, those treated using only the most effective supportive care, or those lost to follow-up. Among the 271 who were refractory to TACE, 108 had been allocated to the sorafenib-conversion group, while 163 continued with TACE (Fig. 1, supplementary Fig.) [5].

Clinical characteristics, demographics, and laboratory tests (Table 1). The cohort involved 229 males (84.5%) and 116 patients (42.8%) from the Menoufia governorate. Depending only on radiological responses, 179 patients (66.1%) were determined to be TACE-refractory, while 92 (33.9%) were considered refractory depending on both radiological and tumour marker responses (Table 1). There were no significant differences in demographic data or clinical characteristics among the sorafenib and TACE-continuation groups (Table 2). However, the sorafenib group had a significantly lower percentage of advanced-stage (BCLC C), disease progression and tumor progression contrasted to the TACE group (*p* < 0.0001) (Table 2).

**Table 1:** Demographic characteristics and presented data of the total studied patients N = 271

		N%
	Mean ± SD N Median (min-max)	62.08 ± 7.251 271 62.00 (39–83)
Gender	Female	42 (15.5%)
	Male	229 (84.5%)
Occupation	Driver	3 (1.1%)
	Employee	66 (24.4%)
	Engineer	2 (0.7%)
	Farmer	18 (6.6%)
	Not work	70 (25.8%)
	Retired	96 (35.4%)
	Teacher	2 (0.7%)
	Worker	14 (5.2%)
Residence	Alexandria	1 (0.4%)
	Behira	60 (22.1%)
	Dakhlia	3 (1.1%)
	Gharbia	48 (17.7%)
	kafr Elsheikh	15 (5.5%)
	Kaliobia	18 (6.6%)
	Matrouh	1(0.4%)
	Menofia	116 (42.8%)
	Shamal Sinai	1 (0.4%)
	Sharkia	8 (3.0%)
Marital Statuus	Married	260 (95.9%)
	Widow	11 (4.1%)
Smoker	No	147 (54.2%)
	Yes	97 (35.8%)
	Ex smoker	27 (10.0%)
Diabetes mellitus	No	249 (91.9%)
	Yes	22 (8.1%)
Hypertension	No	206 (76.0%)
	Yes	65 (24.0%)
viral etiology	No	35 (12.9%)
	Yes	236 (87.1%)
HBsAg	No	271 (100.0%)
Anti-HCV	No	35 (12.9%)
	Yes	236 (87.1%)
DAAS	No	169 (62.4%)
	Yes	102 (37.6%)
non-viral	No	236 (87.1%)
	Yes	35 (12.9%)
ECOG PS	0	250 (92.3%)
	1	21 (7.7%)
No of previous TACE	≤ 2	107 (39.5%)
	> 2	164 (60.5%)
	Mean ± SD Median (min-max)	3.42 ± 1.798 3 (1–9)
cause of refractoriness	Imaging	179 (66.1%)
	TM & Imaging	92 (33.9%)
Bilirubin (mg/dl)	Mean ± SD Median (min-max)	1.021 ± 0.389 1.0 (0.30-2.00)
S. albumin (g/dl)	Mean ± SD Median (min-max)	3.521 ± 0.535 3.6 (0.90–4.90)
International normalized ratio (INR)	Mean ± SD Median (min-max)	1.162 ± 0.231 1.1 (0.90–3.50)
Hemoglobin level (g/dl)	Mean ± SD Median (min-max)	12.756 ± 3.34 12.70 (8.40–44.80)
WBC (X 10^3^/cmm)	Mean ± SD Median (min-max)	5.556 ± 4.164 4.60 (2.00–49.00)
Platelet (X 10^3^/cmm)	Mean ± SD Median (min-max)	135.068 ± 73.515 120.00 (2.60–394.00)
Alanine Aminotransferase (IU/L)	Mean ± SD Median (min-max)	45.800 ± 34.755 34.50 (6.80–144.00)
Aspartate Aminotransferase (IU/L)	Mean ± SD Median (min-max)	59.40 ± 45.19119 45.00 (13.00-190.00)
Urea (mg/dl)	Mean ± SD Median (min-max)	28.632 ± 7.18958 30.00 (18.00–39.00)
S. creatinine (mg/dl)	Mean ± SD Median (min-max)	0.906 ± 0.186 0.90 (0.50–1.40)
Alpha-fetoprotein (ng/ml)	Mean ± SD Median (min-max)	1706.83 ± 10563.034 108.00 (1-155171)
Ascites	No	265 (97.8%)
	Yes	6 (2.2%)
Lesion site	Bilobar	165 (60.9%)
	Unilobar	106 (39.1%)
Lesion number ≤ 3	No	122 (45.0%)
	Yes	149 (55.0%)
Maximum tumor diameter ≤ 5	No	106 (39.1%)
	Yes	165 (60.9%)
Baseline vascular invasion in studied patients	No	271 (100.0%)
Baseline EHMs	No	271 (100.0%)
Baseline advanced stage BCLC in studied patients	No	271 (100.0%)
Baseline CTP of studied patients		
	A (A5&A6)	242 (89.3%)
	B7	29 (10.7%)
Progression to liver dysfunction (CTP C)	No	246 (90.8%)
	Yes	25 (9.2%)
Progression to advanced Stage (BCLC C)	No	151 (55.7%)
	Yes	120 (44.3%)
Disease progression	No	143 (52.8%)
	Yes	128 (47.2%)
Tumor progression (TP)	CR	2 (0.8%)
	PD	195 (72%)
	NA	10 (3.7%)
	PR	8 (3.0%)
	SD	56 (20.6%)
Shift to sorafenib	Continue TACE	163 (60.1%)
	Shifted to sorafenib	108 (39.9%)
Status	Alive	61 (22.5%)
	Dead	173 (63.8%)
	Lost follow up	37 (13.7%)

HBsAg, hepatitis B surface antigen; HCV, hepatitis C virus; DAAS, direct acting antivirals; ECOG PS, Eastern Cooperative Oncology Group Performance Status; EHM, Extrahepatic metastasis; CTP, Child-Turcotte-Pugh; BCLC, Barcelona Clinic Liver Cancer; TACE, Transarterial chemoembolization; Partial response (PR), Stable disease (SD), Progressive disease (PD), Not assessed (NA).

**Table 2:** Demographic characteristics and presented data of the patients in the ‘Shifted to sorafenib’ and ‘continued TACE’ groups

			**Shift to Sorafenib**		Test value	p-value
			No **N = 163**	Yes **N = 108**		
Age	Mean ± SD N Median (min-max)	61.91 ± 7.071 163 61.00 (49–83)		62.33 ± 7.541 108 62.00 (39–81)	t = 0.465	**0.642**
Gender	Female	24 (14.7%)		18 (16.7%)	X2 = 0.187	**0.665**
	Male	139 (85.3%)		90 (83.3%)		
Occupation	Driver	0 (0.0%)		3 (2.8%)	Fisher = 10.793	**0.107**
	Employee	37 (22.7%)		29 (26.9%)		
	Engineer	1 (0.6%)		1 (0.9%)		
	Farmer	15 (9.2%)		3 (2.8%)		
	Not work	45 (27.6%)		25 (23.1%)		
	Retired	55 (33.7%)		41 (38.0%)		
	Teacher	2 (1.2%)		0 (0.0%)		
	Worker	8 (4.9%)		6 (5.6%)		
Residence	Alex	1 (0.6%)		0 (0.0%)	Fisher = 9.153	**0.386**
	Behira	37 (22.7%)		23 (21.3%)		
	Dakhlia	3 (1.8%)		0 (0.0%)		
	Gharbia	28 (17.2%)		20 (18.5%)		
	kafr Elsheikh	11 (6.7%)		4 (3.7%)		
	Kaliobia	13 (8.0%)		5 (4.6%)		
	Matrouh	0 (0.0%)		1 (0.9%)		
	Menofia	67 (41.1%)		49 (45.4%)		
	Shamal Sinai	0 (0.0%)		1 (0.9%)		
	Sharkia	3 (1.8%)		5 (4.6%)		
Marital Status	Married	158 (96.9%)		102 (94.4%)	X2 = 1.033	**0.31**
	Widow	5 (3.1%)		6 (5.6%)		
Smoker	No	87 (53.4%)		60 (55.6%)	X2 = 1.312	**0.519**
	Yes	57 (35.0%)		40 (37.0%)		
	Ex smoker	19 (11.7%)		8 (7.4%)		
Diabetes mellitus	No	146 (89.6%)		103 (95.4%)	X2 = 2.929	**0.087**
	Yes	17 (10.4%)		5 (4.6%)		
Hypertension	No	129 (79.1%)		77 (71.3%)	X2 = 2.19	**0.139**
	Yes	34 (20.9%)		31(28.7%)		
viral etiology	No	21 (12.9%)		14 (13.0%)	X2 = 0.0001	**0.985**
	Yes	142 (87.1%)		94 (87.0%)		
HBsAg	No	163 (100.0%)		108 (100.0%)	-	**-**
Anti-HCV	No	21 (12.9%)		14 (13%)	X2 = 0.2	**0.65**
	Yes	142 (87.1%)		94 (87%)		
DAAS	No	107 (65.6%)		62 (57.4%)	X2 = 1.8	**0.171**
	Yes	56 (34.4%)		46 (42.6%)		
non-viral	No	142 (87.1%)		94 (87%)	X2 = 295	**0.587**
	Yes	21 (12.9%)		14 (13.0%)		
ECOG PS	0	152 (93.3%)		98 (90.7%)	X2 = 0.573	**0.449**
	1	11 (6.7%)		10 (9.3%)		
No of previous TACE	1	16 (9.9%)		12 (11.3%)	Fisher 4.89	**0.796**
	2	52 (32.1%)		27 (25.5%)		
	3	31 (19.1%)		19 (17.9%)		
	4	25 (15.4%)		14 (13.2%)		
	5	18 (11.1%)		15 (14.2%)		
	6	12 (7.4%)		12 (11.3%)		
	7	3 (1.9%)		3 (2.8%)		
	8	5 (3.1%)		3 (2.8%)		
	9	0 (0.0%)		1 (0.9%)		
	Mean ± SD	3.31 ± 1.724		3.58 ± 1.902		
	Median (min-max)	3 (1–8)		3 (1–9)		
cause of refractoriness	Imaging	114 (69.9%)		65 (60.2%)	X2 = 2.756	**0.097**
	TM & Imaging	49 (30.1%)		43 (39.8%)		
Bilirubin (mg/dl)	Mean ± SD	1.0460 ± 0.379		0.983 ± 0.405	T = 268	**0.196**
	Median (min-max)	1.0 (0.30-2.00)		0.90 (0.40-2.00)		
S. albumin (g/dl)	Mean ± SD	3.457 ± 0.543		3.618 ± 0.51080	U = 2.46	**0.014**
	Median (min-max)	3.5 (0.90–4.70)		3.6 (1.99–4.90)		
International normalized ratio (INR)	Mean ± SD	1.171 ± 0.268		1.148 ± 0.159	U = 0.007	**0.994**
	Median (min-max)	1.1 (0.90–3.50)		1.1 (0.90–1.70)		
Hemoglobin level (g/dl)	Mean ± SD	12.511 ± 2.839		13.097 ± 3.914	U = 1.537	**0.124**
	Median (min-max)	12.30 (9.00–38.00)		13.0 (8.40–44.80)		
WBC (X 10^3^/cmm)	Mean ± SD	5.422 ± 4.41		5.741 ± 3.812	T = 213	**0.581**
	Median (min-max)	4.6 (2.00–49.00)		4.85 (2.00–35.00)		
Platelet t (X 10^3^/cmm)	Mean ± SD	127.289 ± 64.253		145.70 ± 83.763	U = 1.364	**0.173**
	Median (min-max)	116.00 (2.60–368.00)		135.50 (14.00-394.00)		
Alanine Aminotransferase (IU/L)	Mean ± SD Median (min-max)	54.548 ± 39.984 39.00 (11.00-140.00)		39.773 ± 29.628 34.00 (6.80–144.00)	U = 1.38	**0.168**
Aspartate	Mean ± SD	65.968 ± 48.129		54.773 ± 42.958	T = 73	**0.294**
Aminotransferase (IU/L)	Median (min-max)	45.00 (13.00-175.00)		45.00 (14.00-190.00)		
Urea (mg/dl)	Mean ± SD	28.700 ± 7.103		28.556 ± 7.715	T = 16.38	**0.967**
	Median (min-max)	29.500 (18.00–38.00)		30.00 (18.00–39.00)		
S. creatinine (mg/dl)	Mean ± SD	0.921 ± 0.201		0.8915 ± 0.170	T = 115	**0.398**
	Median (min-max)	0.90 (0.50–1.40)		0.90 (0.50–1.20)		
Alpha-fetoprotein (ng/ml)	Mean ± SD	1051.77 ± 3548.611		2689.42 ± 16129.499	U = 0.204	**0.839**
	Median (min-max)	84.00 (1-33108)		142.50 (2-155171)		
Ascites	No	158 (96.9%)		107 (99.1%)	Fisher = 1.376	**0.407**
	Yes	5 (3.1%)		1 (0.9%)		
Lesion site	Bilobar	107 (65.6%)		58 (53.7%)	X2 = 3.889	**0.049**
	Unilobar	56 (34.4%)		50 (46.3%)		
Lesion number ≤ 3	No	78 (47.9%)		44 (40.7%)	X2 = 1.327	**0.249**
	Yes	85 (52.1%)		64 (59.3%)		
Maximum tumor diameter ≤ 5	No	67 (41.1%)		39 (36.1%)	X2 = 68	**0.41**
	Yes	96 (58.9%)		69 (63.9%)		
Baseline vascular invasion in studied patients	No	163 (100.0%)		108 (100.0%)	-	**-**
Baseline EHMs	No	163 (100.0%)		108 (100.0%)	-	**-**
Baseline advanced stage BCLC in studied patients	No	163 (100.0%)		108 (100.0%)	-	**-**
Baseline CTP of studied patients					X2 = 1.05	0.304
	A (A5& A6)	143(87.7%)		99(91.7%)		
	B7	20 (12.3%)		9 (8.3%)		
Progression to liver dysfunction (CTP C)	NO	144 (88.3%)		102 (94.4%)	X2 = 2.88	**0.89**
	Yes	19 (11.7%)		6 (5.6%)		
Progression to advanced Stage (BCLC C)	No	77 (47.2%)		74 (68.5%)	X2 = 11.9	**0.001**
	Yes	86 (52.8%)		34 (31.5%)		
Disease progression	No	71 (43.6%)		72 (66.7%)	X2 = 13.918	**0.0001**
	Yes	92 (56.4%)		36 (33.3%)		
Tumor progression (TP)	CR	0 (0.0%)		2 (1.8%)	Fisher = 23.8	**0.0001**
	PD	132 (81%)		63 (58.3%)		
	NA	1 (0.6%)		9 (8.3%)		
	PR	2 (1.2%)		6 (5.6%)		
	SD	28 (17.2%)		28 (26.0%)		
Status	Alive	14 (9.7%)		47 (52.2%)	X2 = 51.9	**0.0001**
	Dead	130 (90.3%)		43 (47.8%)		

**Fig. 2:**
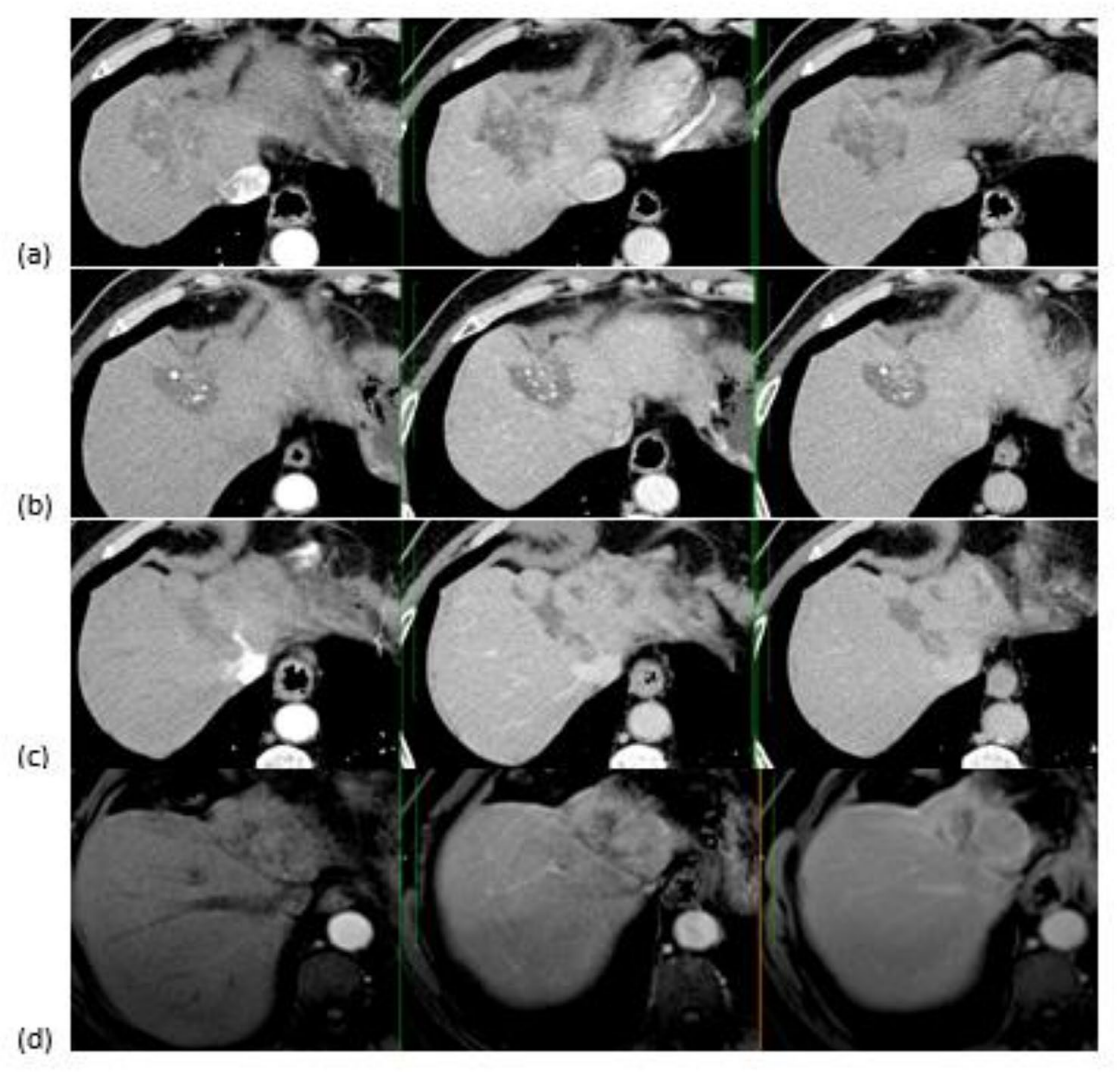
A case of male patient in the seventh decade of life with left lobe HCC (**a**) Triphasic CT before management demonstrating arterial enhancement & delayed washout within the lesion. (**b**) Follow-up triphasic CT after TACE revealed no residual activity within the managed lesion. (**c**) Follow-up triphasic CT 12 months after TACE revealed newly developed left lobe infiltrative HCC. (**d**) Patient received Sorafinib and follow-up dynamic MRI after 6 months revealed atrophy of the left lobe with complete tumor resolution & no residual or recurrent activity. In subsequent follow-ups on sorafenib, he continued to show a complete response

Within the sorafenib group, a total of 36 individuals maintained their sorafenib treatment to the point of disease progression, However, 17 patients discontinued due to severe adverse effects. Specifically, 9 patients developed severe hand-foot syndrome, 1 patient with cardiac issues could not tolerate the treatment, 5 patients experienced a significant deterioration in liver function, and 2 patients suffered from variceal bleeding. According to mRECIST criteria, 2 patients had a complete response (CR) (Figs. 2), 6 had partial response (PR), 28 exhibited stable disease (SD), 63 exhibited progressive disease (PD), and 9 had been not assessed (NA). The median TTP with sorafenib therapy was 12.167 months (95% CI, 9.446–14.888). In the TACE group, 163 participants were included after being considered TACE-refractory. According to mRECIST criteria, 2 patients exhibited PR, 28 exhibited SD, 132 exhibited PD, and 1 had been NA (Table 2). The median TTP with TACE following being determined to be unresponsive to TACE was 6.067 months (95% CI, 5.519–6.614) (Table 3).

**Table 3:** Kaplan-Meier survival mean and median of the presented data of the patients in the ‘Shifted to sorafenib’ and ‘continued TACE’ groups:

		N of Events	Meana				Median				Log Rank (Mantel-Cox)	p-value
			Estimate	Std. Error	**95%) Confidence Interval**		Estimate	Std. Error	**95%) Confidence Interval**			
					Lower Bound	Upper Bound			Lower Bound	Upper Bound		
Survival	Total survival	173	19.349	0.868	17.649	21.050	16.233	0.884	14.501	17.966		
Survival	TACE	130	15.485	0.703	14.107	16.863	14.200	0.643	12.939	15.461		
	Shift to SORAFENIB	43	27.353	1.915	23.599	31.107	25.300	3.687	18.074	32.526	36.893	**0.0001**
	Overall		19.349	0.868	17.649	21.050	16.233	0.884	14.501	17.966		
Liver dysfunction (child c)	TACE	19	26.597	1.658	23.347	29.847	30.467	4.511	21.625	39.308		
	Shift to SORAFENIB	6	37.787	1.797	34.265	41.308	.	.	.	.	12.251	**0.0001**
	Overall	25	33.772	1.609	30.617	36.926	.	.	.	.		
Advanced stage progression (BCLC C)	TACE	86	14.637	1.002	12.673	16.601	11.167	0.732	9.732	12.601		
	Shift to SORAFENIB	34	25.909	1.819	22.343	29.475	23.367	2.136	19.179	27.554	31.130	**0.0001**
	Overall	120	19.699	1.135	17.475	21.923	18.333	2.330	13.766	22.901		
Disease progression	TACE	92	14.059	0.924	12.247	15.870	11.167	0.757	9.682	12.651		
	Shift to SORAFENIB	36	25.154	1.754	21.716	28.592	23.367	1.686	20.063	26.671	34.154	**0.0001**
	Overall	128	18.804	1.033	16.781	20.828	18.233	2.546	13.243	23.224		
Tumor progression	TACE	131	7.868	0.544	6.801	8.934	6.067	0.279	5.519	6.614		
	Shift to SORAFENIB	63	16.027	1.391	13.300	18.754	12.167	1.388	9.446	14.888	43.257	**0.0001**
	Overall	194	11.150	0.719	9.740	12.559	8.167	0.501	7.186	9.148		

**Fig. 3:**
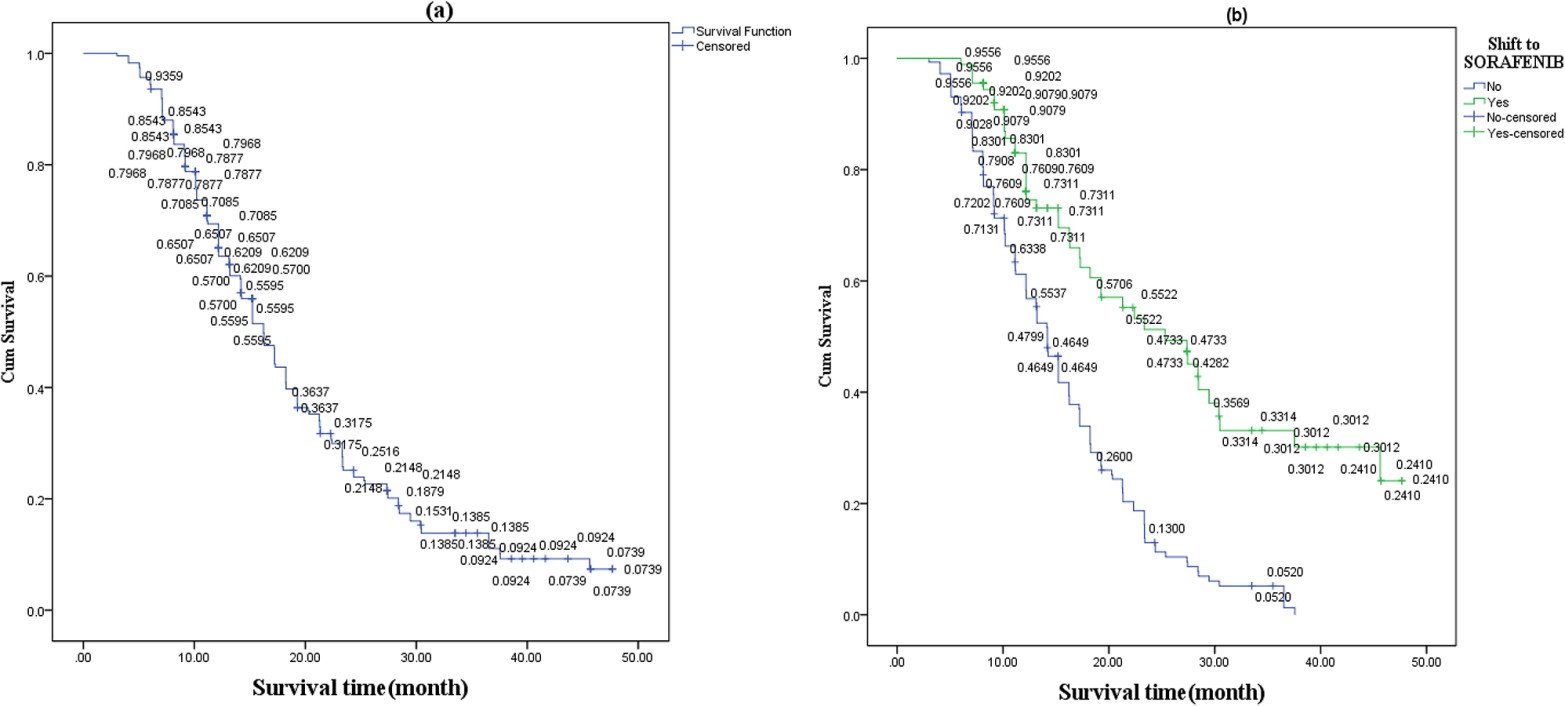
Kaplan-Meier curves (**a**) Total survival time, (**b**) Survival time of conversion to the sorafenib group (green line) and the continued TACE group (blue line)

**Fig. 4:**
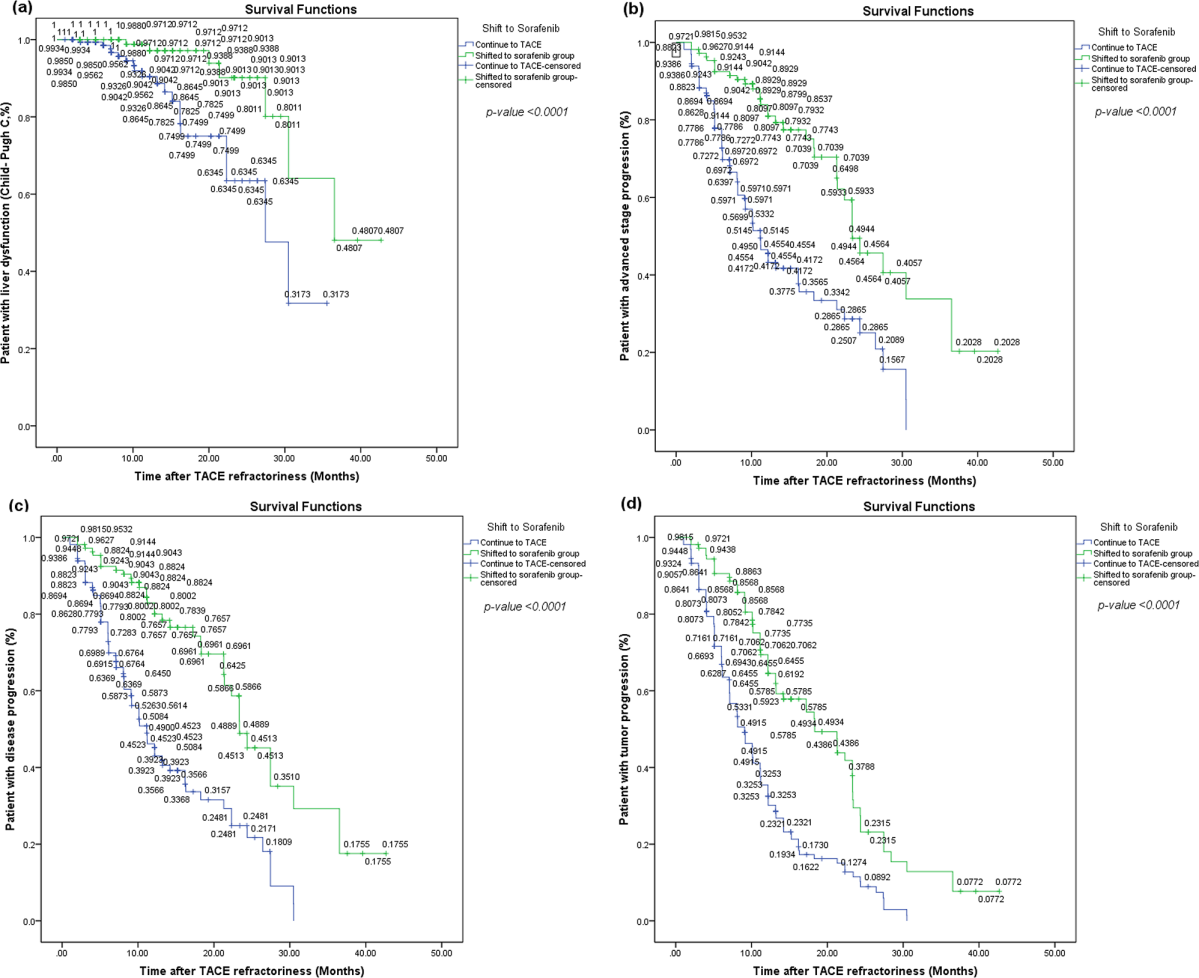
Kaplan-Meier curves of the time to liver dysfunction (**a**), time to advanced stage progression (**b**), Time to disease progression (**c**) and time to tumor progression (**d**). The green lines indicate the conversion to the sorafenib group, and the blue lines indicate the continued TACE group

Out of the 271 individuals analyzed, 173 experienced mortalities throughout the research duration, 61 survived, and 37 could not be tracked for further observation. The median duration of follow-up was 12.4 months, as seen in (Table 3; Fig. 3). The sorafenib group had a considerably greater median OS compared to the TACE group [25.30 months (95% CI, 18.074–32.526) vs. 14.2 months (95% CI, 12.939–15.461); *p* = 0.0001] (Table 3; Fig. 4). The mean time to liver dysfunction in the sorafenib group was 37.787 months (95% CI, 34.265–41.308), while the mean and median times in the TACE group had been 26.597 months (95% CI, 23.347–29.847) and 30.46 months (95% CI, 21.625–39.308), respectively. The median durations for advancing to the advanced stage in the sorafenib and TACE groups were 23.367 months (95% CI, 19.179–27.554) and 11.167 months (95% CI, 9.732–12.601), respectively. The duration till hepatic failure and advanced stage were significantly reduced in the TACE group compared to the sorafenib group (*p* < 0.0001). The TACE group also exhibited a markedly reduced median TTDP compared to the sorafenib group (*p* < 0.001). (Table 3; Fig. 4)

Survival following individuals were deemed TACE-refractory was examined using both univariate (UV) and multivariate (MV) analysis (Table 4). UV analyses determined several significant factors contributing to survival: ECOG PS, serum albumin, AFP levels, switching to sorafenib, advanced stage BCLC C, liver dysfunction (CTP C), tumor progression, and disease progression. In the MV analysis, important independent prognostic factors included AFP level, advanced stage BCLC C, and conversion to sorafenib.

**Table 4:** Univariate and multivariate survival analyses

	Univariate cox regression					multivariate cox regression				
	B	Exp(B) HR	95.0% CI for Exp(B)		p-value	B	Exp(B) HR	95.0% CI for Exp(B)		p-value
			Lower	Upper				Lower	Upper	
Age	0.025	1.026	0.991	1.062	**0.151**					
Gender	0.139	1.149	0.803	1.642	**0.448**					
Diabetes mellitus	0.585	1.795	0.885	3.640	**0.105**					
Hypertension	− 0.028	0.972	0.723	1.307	**0.852**					
Anti HCV	0.231	1.260	0.888	1.788	**0.196**					
DAAS	− 0.118	0.889	0.518	1.526	**0.669**					
ECOG PS	-1.104	0.332	0.139	0.793	**0.013**					
Bilirubin	− 0.365	0.694	0.330	1.461	**0.337**					
S. albumin	− 0.331	0.718	0.549	0.940	**0.016**	0.101	1.106	0.706	1.733	0.659
Platelet	0.002	1.002	0.999	1.005	**0.258**					
Creatinine	-1.373	0.253	0.053	1.217	**0.086**					
Alpha-fetoprotein	0.000	1.00001	1.000	1.001	**0.007**	0.000	1.001	1.000	1.0003	0.049
Ascites	1.530	4.617	0.178	119.487	**0.357**					
Lesion site	− 0.223	0.800	0.622	1.030	**0.083**					
Lesion number ≤ 3	0.037	1.038	0.599	1.798	**0.895**					
Shift to sorafenib	-1.135	0.321	0.173	0.598	**0.000**	− 0.925	0.397	0.200	0.786	0.008
Progression to advanced Stage BCLC C	1.558	4.749	2.451	9.203	**0.000**	0.922	2.514	1.085	5.828	0.032
Liver dysfunction CTP C	1.391	4.019	1.250	12.928	**0.020**	0.572	1.771	0.515	6.085	0.364
Tumor progression	1.159	3.187	1.915	5.302	**0.0001**	0.381	1.464	0.741	2.891	0.273
Disease progression	1.615	5.029	2.652	9.535	**0.000**					

HR (hazard ratios)

## Discussion

TACE is the established treatment for individuals with intermediate-stage HCC who have Child-Pugh class A or B function of the liver and numerous intrahepatic lesions, but no vascular infiltration or extrahepatic metastasis [6, 14, 15]. Although TACE may successfully provide localized tumour control, it often necessitates repeated applications, leading to a gradual decline in liver functioning, decrease tumour necrotic effects, the emergence of new lesions, and a persistent elevation in tumor markers [5, 8, 22]. Consequently, the concept of TACE refractoriness or failure, indicative of poor response, has garnered significant attention in recent years [13, 23, 24]. This concept was first introduced by the JSH in 2010 and updated in 2014 [6, 7]. Subsequently, TACE refractoriness or failure has been recognized in various therapy guidelines, including those by the EASL [16, 17], the AASLD [14, 24], the European Society for Medical Oncology (EPOIHCC) [9], and the APASL [15]. However, the criteria for defining TACE refractoriness or failure remain controversial [13, 25].

Repeated TACE among individuals who no longer respond to the procedure’s results in reduced functioning of the liver and a negative prognosis [5, 26]. Therefore, it is advisable to explore alternate therapies, such as systemic therapy [6, 17, 24, 26]. Switching from TACE to systemic therapy such as sorafenib is generally considered after 2–3 cycles of TACE, especially when imaging (e.g., CT or MRI) shows evidence of progressive disease or inadequate response [5–7, 24, 25]. The timing of switching from TACE should be personalized, based on imaging results, liver function, and the patient’s overall condition. Significant tumor progression or insufficient response after a certain number of TACE sessions may indicate the need for an alternative treatment strategy.

Shifting to sorafenib following TACE resistance is very probable to maintain the liver’s function and decrease the occurrence of disease progression events, like extrahepatic spread or vascular infiltration [5, 6, 9, 16, 17, 24]. While sorafenib has been a cornerstone in treating advanced HCC, newer systemic therapies and immune checkpoint inhibitors (ICIs) offer additional options. Lenvatinib, another multi-kinase inhibitor, has shown comparable efficacy to sorafenib in first-line treatment and serves as a viable alternative with a broader target range [27]. Cabozantinib, targeting MET, VEGFR2, and AXL, provides significant benefits in progression-free and overall survival in patients who have failed previous treatments, though it may cause more gastrointestinal side effects [28]. Regorafenib, effective for those who have progressed on sorafenib, improves OS but is associated with fatigue and hand-foot syndrome [29]. In recent years, ICIs, such as atezolizumab and nivolumab, have emerged as promising options. Atezolizumab, often combined with bevacizumab, has demonstrated superior efficacy in terms of PFS and OS compared to sorafenib in the first-line setting. ICIs generally have a different side effect, including immune-related adverse events, which can be manageable with appropriate monitoring and treatment [30].

This retrospective work had been performed through a medical record review of our intermediate-stage HCC individuals who had become TACE-refractory. The purpose of this work was comparing the clinical outcomes of sorafenib shifting with those of ongoing TACE. The study predominantly included male individuals with a mean age of 62 years. This aligns with findings by Bengtsson et al., indicating that HCC is more prevalent in males and tends to occur in older individuals, particularly those with cirrhosis [31].

In terms of assessing the response to therapy for both groups using mRECIST criteria [21], our results demonstrated that sorafenib was effective in prolonging both OS and TTP. The median TTP and OS were significantly prolonged in the sorafenib group comparing with the group that continued TACE (12.16 vs. 6.06 months for TTP, and 25.3 vs. 16.2 months for OS, respectively; p-value = 0.0001). Similarly, previous studies have also shown the efficiency of sorafenib in prolonging OS and TTP [5, 32]. For instance, Ogasawara et al. performed a comparison between the usage of sorafenib and TACE. They discovered that both OS and TTP were substantially greater in the sorafenib group (SG) comparing with the TACE group (TG). The OS was 25.4 months in the SG and 11.5 months in the TG, while the TTP was 22.3 months in the SG and 7.7 months in the TG [5]. Similarly, research conducted by Arizumi et al. found that the OS in the SG was 24.7 months, whereas in the TG it was 13.6 months [18].

Our analyses showed that the duration before reaching an advanced stage was notably prolonged in the sorafenib switching group comparing with the ongoing TACE group (p-value = 0.0001). Additionally, we found that ongoing TACE significantly reduced the duration to liver failure compared to conversion to sorafenib therapy (p-value = 0.0001). This finding aligns with previous reports indicating that frequent TACE on an intensive schedule increase the risk of liver dysfunction [5, 33, 34]. Consequently, we concluded that patient response to TACE worsened after they were considered TACE-refractory.

The time to disease progression (TTDP) was significantly lengthened in the sorafenib switching group comparing with the ongoing TACE group, with median times of 23.36 months versus 11.16 months, respectively (p-value = 0.0001). Similarly, Ogasawara et al. found that TTDP was prolonged by switching to sorafenib in intermediate-stage HCC patients after confirming TACE refractoriness [5].

Given that repeated TACE procedures in individuals unresponsive to treatment have led to deteriorated liver function and unsatisfactory response, thereby significantly impacting survival, our univariate and multivariate analyses underscore the importance of switching TACE-refractory individuals to sorafenib. Despite this, the optimal timing for shifting from TACE to sorafenib in intermediate-stage HCC individuals remains controversial [5, 13, 25].

Our study had several limitations, including potential selection and information biases due to its single-site, retrospective nature. Although we ensured comparability between groups using observed characteristics, potential unmeasured confounders may still affect the results. We did not apply Propensity Score Matching (PSM), which could have further reduced selection bias; however, our existing methods showed adequate balance. Additionally, treatment protocols for TACE vary significantly between institutions and regions. Therefore, prospective multicenter randomized studies are crucial to ascertain the clinical effectiveness of sorafenib switching and determine the most favorable moment to switch from TACE to sorafenib, and develop a universally accepted definition of TACE refractoriness.

## Conclusion

Conversion to sorafenib may improve outcomes, as evidenced by prolonged OS, TTP and TTDP in patients who have not responded to TACE treatment with intermediate-stage HCC.

## Electronic supplementary material

Below is the link to the electronic supplementary material.

Supplementary Material 1

## Data Availability

Accessible upon inquiry from the corresponding author.

## Declarations

### Data sharing statement

No other data are available.

## Abbreviations

AASLD: American Association for the Study of Liver Diseases
APASL: Asian Pacific Association for the Study of the Liver
AFP: Alpha Fetoprotein
BCLC: Barcelona Clinic Liver Cancer
CI: Confidence interval
CT: Computed tomography
CTP: Child-Pugh Class
CR: Complete response
EASL: European Association for the Study of the Liver
HCC: Hepatocellular Carcinoma
ICIs: Immune checkpoint inhibitors
IRB: Institutional Review Board
JSH: Japan Society of Hepatology
MRI: Multivariate
OS: Overall Survival
PD: Progressive disease
PR: Partial response
SD: Stable disease
TACE: Transarterial Chemoembolization
TTP: The time to tumor progression
TTDP: The time to disease progressions
The time to disease progression: Univariate
