# Supplementary figures and images for: Effectiveness of sorafenib in treating intermediate-stage hepatocellular carcinoma patients refractory to transarterial chemoembolization
Source: BMC Cancer. 2024 Nov 28;24:1466. doi: 10.1186/s12885-024-13199-1 (PMC11603851; doi:10.1186/s12885-024-13199-1)

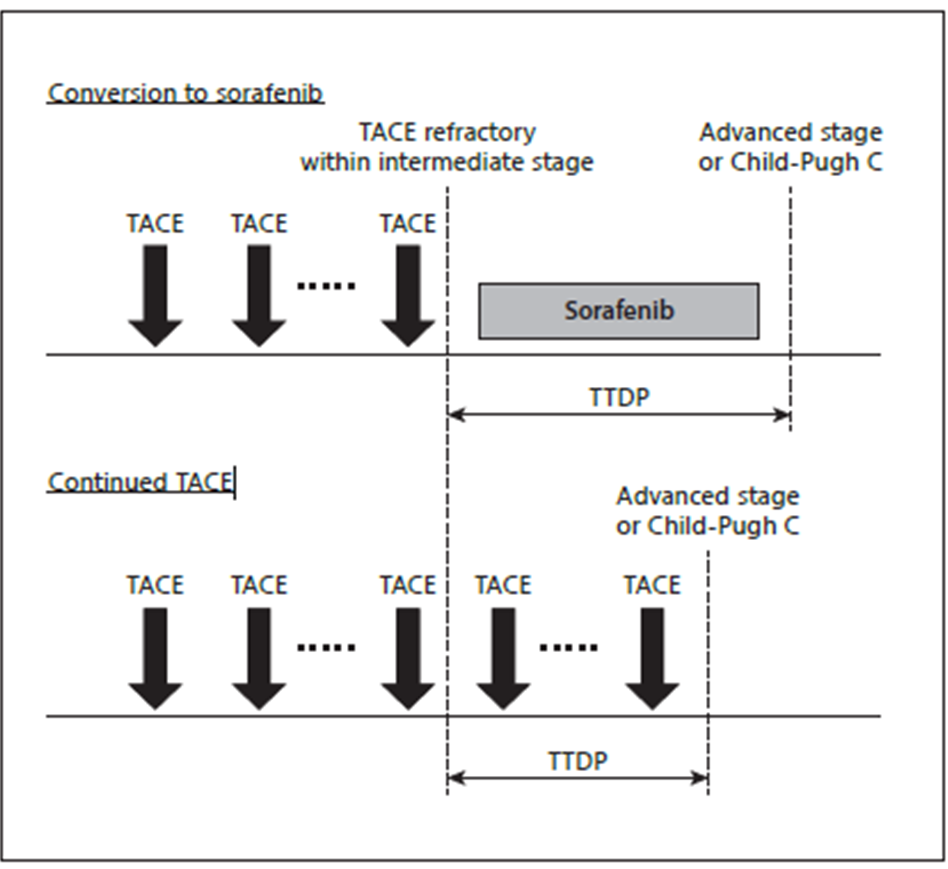


**Supplementary Figure**: Overview of the clinical course of enrolled patients, **Ogasawara et al, 2014 [5].**

Supplement: Supplementary file 1 — Supplementary Material 1 [file 12885_2024_13199_MOESM1_ESM.docx]
